# Artificial Intelligence-Based Hospital Malnutrition Screening: Validation of a Novel Machine Learning Model

**DOI:** 10.1055/a-2635-3158

**Published:** 2025-11-14

**Authors:** Adam M. Bernstein, Pierre Janeke, Richard V. Riggs, Emily Burke, Jemima Meyer, Meagan F. Moyer, Keiy Murofushi, Raymond A. Botha, Josiah El Michael Meyer

**Affiliations:** 1 HealthLeap, Inc., San Francisco, California, United States; 2 Department of Physical Medicine and Rehabilitation, Cedars-Sinai Medical Center, West Hollywood, California, United States

**Keywords:** malnutrition, screening, artificial intelligence, hospital, validation

## Abstract

**Background:**

Despite its morbidity, mortality, and financial burden, in-hospital malnutrition remains underdiagnosed and undertreated. Artificial intelligence (AI) offers a promising clinical informatics solution for identifying malnutrition risk and one that can be coupled with clinician-delivered patient care.

**Objectives:**

The objectives of the study were to evaluate an AI-based hospital malnutrition screening model in a large and diverse inpatient population and to compare it to the currently used clinician-delivered malnutrition screening tool.

**Methods:**

We studied the performance of a gradient-boosted decision tree model incorporating a large language model (LLM) for feature extraction using the electronic medical record data of 106,449 patients over 3.75 years.

**Results:**

The model's area under the receiver operating curve was 0.92 (95% confidence interval [CI]: 0.91–0.92) on the first day of hospitalization and rose to 0.95 (95% CI: 0.95–0.96) using the maximum risk predicted for each patient throughout hospitalization, indexed against discharge-coded malnutrition. Similar results were observed when indexed against dietitian-recorded malnutrition. The model outperformed the nurse-administered, modified version of the Malnutrition Screening Tool (MST) that was used in practice. Patients identified by the model had higher likelihoods of readmission and death compared with patients identified by the nurse-administered screener.

**Conclusion:**

Our study findings provide validation for a novel model's use in the prediction of in-hospital malnutrition.

## Background and Significance

Hospitals today face the challenge of identifying and treating malnutrition, a condition that is underdiagnosed and undertreated, yet may have severe and fatal consequences. 1 2 The condition is an acute, subacute, or chronic state in which varying degrees of overnutrition or undernutrition with or without inflammation lead to changes in body composition and function. 3 Undernutrition, or protein-calorie malnutrition, the most common type of malnutrition in the hospital 4 and the focus of the following research, may portend higher morbidity and mortality, longer lengths of hospitalization, and heightened healthcare costs. 5 Yet, despite broad efforts, between 20 and 50% of patients are estimated to be malnourished, and less than half of patients either at risk of, or diagnosed with malnutrition, receive optimal nutritional care. 5 6 7 Those who do receive both a prompt diagnosis and multidisciplinary, nutrition-focused care have improved quality of life, fewer health complications, and less frequent rehospitalizations. 8

Screening for hospital malnutrition, however, remains a systemic challenge. 9 The Joint Commission mandates that screening be completed within 24 hours of a patient's hospital admission, 10 and yet with over 50 malnutrition screening instruments of varying performance, none is universally adopted. 11 12 The Academy of Nutrition and Dietetics, the world's largest association of food and nutrition practitioners, 13 recently reviewed the six most-studied malnutrition screening tools—including the Malnutrition Screening Tool (MST), Malnutrition Universal Screening Tool, Mini Nutritional Assessment-Short Form, Short Nutritional Assessment Questionnaire, Mini Nutritional Assessment-Short Form Body Mass Index, and Nutrition Risk Screening 2002—and concluded that none had high validity, reliability, and strong supportive evidence. 14 The Academy took the position that, based upon current evidence, the MST should be used to screen adults for malnutrition (undernutrition), regardless of age, medical history, or setting. 14 Of note, nutrition-related biomarkers, such as serum proteins, have proven unreliable as they are influenced by inflammation, a common condition in the acutely ill patient. 15 16 Artificial intelligence (AI) initiatives have recently been undertaken to assist, although these too are of varying performance and limited deployment. 17

Collectively, this prior work demonstrates a place for novel, evidence-based, unbiased, accessible, and scalable clinical informatics solutions to help identify hospital malnutrition risk.

## Objective

The following study was undertaken to evaluate an AI-based hospital malnutrition screening model as well as the inclusion of a large language model (LLM) for analyzing clinical notes, an approach that has not been done in the hospital for this purpose to date. The hypothesis was that the model would demonstrate better performance than the currently used hospital screening method, a nurse-administered, modified version of the Malnutrition Screening Tool (M-MST). 18 The MST asks two questions (1. “Have you recently lost weight without trying?” [and if yes, “How much weight have you lost?”] and 2. “Have you been eating poorly because of a decreased appetite?”) and provides a score based on responses (within a range of 0–5). A score of 2 or more suggests that the patient is at risk of malnutrition. By contrast, with the M-MST, patients who answered positively to either of the two MST questions were referred to a registered dietitian for evaluation. Patients who were neither referred to a dietitian by screening nor referred to a dietitian by consultation request were re-screened at set intervals, such as weekly, thereafter. This study's model, by comparison, could screen every hospitalized patient every day.

## Methods

### Study Cohort and Data Source

**Fig. 1:**
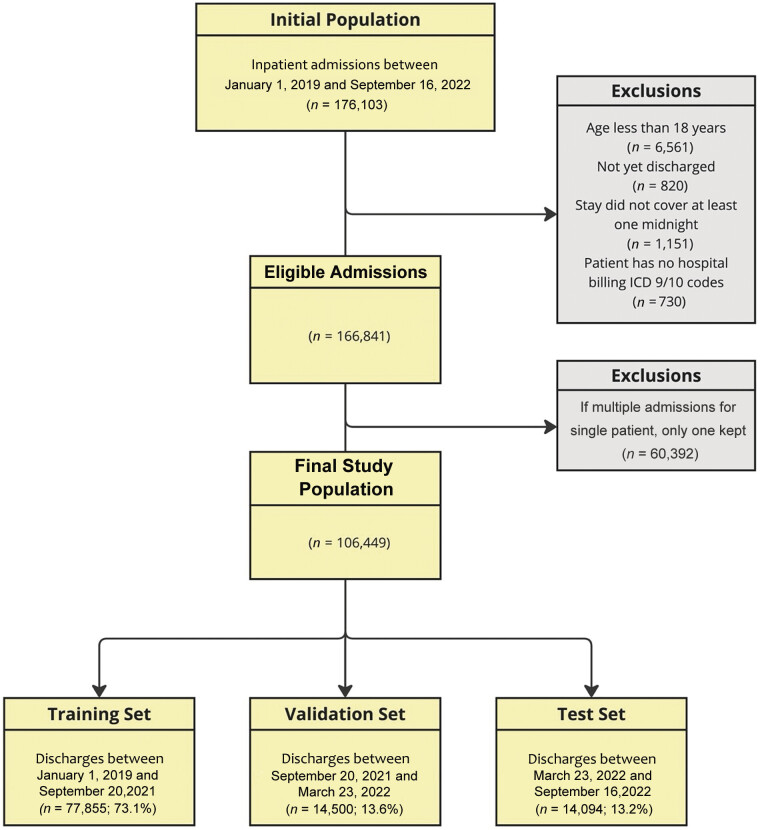
Study flow of participants.

All adults 18 years of age and older admitted to the Cedars-Sinai Medical Center between January 1, 2019, and September 14, 2022, were eligible for inclusion in this retrospective study. The study site is one of the largest non-profit academic medical centers in the United States, with nearly 900 beds and over 2,000 physicians, and is responsible for providing a range of clinical services, including complex care treatment. 19 While pediatric patients may suffer from malnutrition, they were not included in this study as the focus was on the adult population. The MST is not a screening tool typically used for pediatric populations, and the exclusion of pediatric patients allowed for certain types of feature engineering (e.g., trends in body mass index [BMI]). De-identified data for each patient were pulled from the earliest available date in the electronic health record (EHR) through the time of discharge. Sample size and power estimates were not conducted; however, the estimated minimum sample size required for a screening study ranges from 22 (prevalence = 90%, H _o_ = 0.5 and H _a_ = 0.8) to 980 (prevalence = 5%, H _o_ = 0.5 and H _a_ = 0.7), and our study population was much larger. 20 Patients were excluded if they did not stay overnight in the hospital, did not have at least one hospital billing code, or were not yet discharged. The final study population included 166,841 admissions, including 106,449 unique patients ( Fig. 1 ).

### Model Evaluation

The model's performance was evaluated against binary variables indicating whether malnutrition was recorded during hospitalization or coded in a patient's medical chart at hospital discharge. Dietitians recorded malnutrition during hospitalization using the recently validated ASPEN and AND Indicators for Malnutrition (AAIM) diagnostic tool. 21 22 Physicians recorded malnutrition in their notes and/or by using International Classification of Diseases (ICD)-9 or ICD-10 codes during admission or upon discharge. 23 For the analysis, ICD-9-CM version 32 (captured in the EHR from past hospitalizations) and ICD-10-CM and ICD-10-PCS (which had stable malnutrition codes across versions) were used. Hospital coding teams added billing codes based on physician documentation for malnutrition and/or cachexia, coded as 799.4 (ICD9) or R64 (ICD10), and both were used for model evaluation. To avoid target leakage, nutritional interventions during hospitalization were not included in the model. 24

### Training, Validation, and Test Sets

A supervised machine learning gradient-boosted decision tree probabilistic classifier, incorporating an LLM for feature extraction, was built to predict the risk of malnutrition. The classifier was chosen for reasons such as its performance, computation time, and ability to accommodate an extensive number of features and interactions. 25 26 Input data included structured, semistructured (e.g., wound care data), and unstructured (e.g., clinician notes) data, with semistructured and unstructured data converted into structured data ( Supplementary Fig. S1 [available in the online version only]). The model generated a risk score for malnutrition for each hospitalized patient each day (between 0 and 100, where 0 meant no risk and 100 was very high risk). One eligible admission per patient was randomly selected and placed into training, validation, or test sets using an Out-of-Time (OOT) sampling method based on discharge time. Unique patients in the training set totaled 77,855, while the validation and test sets contained 14,500 and 14,094, respectively.

Model inputs were extracted from the following groups of data: demographic (e.g., date of birth and sex); health status (e.g., weight, height, BMI, laboratory values, admission diagnosis, problem list, clinical conditions, allergies, and medications); health care delivery data (e.g., hospital billing codes, professional billing codes, and prior admission ICD codes); and hospital stay data (e.g., clinician notes, input and output flowsheet data, food and nutrition flowsheets, nutrition orders, lines and drains, and airways). For food and nutrition flowsheets, we looked at nutrition intake data and avoided target leakage by not including data on nutrition support (enteral and parenteral nutrition) or oral nutrition supplementation interventions. The M-MST was not included as a feature.

Multiple rounds of feature selection began with training separate models with ICD codes, laboratory values, and medications to identify features of highest importance. Feature engineering followed, which allowed for creating a final feature set from the initial one, and included, for instance, evaluating feature importance, laddering child ICD codes up to parent codes, especially in instances of infrequency (e.g., less than 0.1% prevalence), using occurrence counts over defined time intervals, and assessing linear trends within time intervals.

Missing data were imputed using the default LightGBM method with the imputation model fit on the training set and applied to the training, validation, and test sets. 27

### Large Language Model

We wrote 20 questions with multiple-choice answers, informed by AAIM, the recently validated Global Leadership Initiative on Malnutrition (GLIM) criteria, and current malnutrition research ( Supplementary Table S1 [available in the online version only]), and then used OpenChat-3.5-0106 to query clinician notes. 28 29 30 31 32 33 34 We had previously observed that OpenChat-3.5-0106, a fine-tuned version of Mistral 7B, had an average accuracy of 63.20 and 73.7% on the medical benchmarking datasets and QuALITY datasets, respectively. 35 36 37 Accuracy degraded after a threshold of approximately 4,000 tokens ( Supplementary Tables S2 and S3 [available in the online version only]). For the study, we selected the longest clinician note of any type for each patient from 24 hours prior to admission until the first midnight on the day of admission. Each note had at least 500 tokens, equivalent to approximately 400 words, and 162,507 (97.4%) of admissions met the selection criteria. With each note, the model was queried and allowed to respond with one of the predefined multiple-choice answers. Key-value caching and constrained token generation were used to produce discrete answers with high throughput and lower computational requirements. The resulting question–answer pairs were incorporated as model features. We observed that when LLM features alone were added to the model, they performed well, but not as well as other model features alone or other model features plus LLM features combined ( Supplementary Table S4 [available in the online version only]). The final model included LLM features plus other model features combined.

### Statistical Analysis

Descriptive statistics of admissions were generated using median and interquartile range or number and percentage, stratified by the presence of coded malnutrition. For continuous variables, Welch's *t* -test was used, while Fisher's exact test was used for categorical variables. Model performance was evaluated by sensitivity, specificity, positive predictive value (PPV), and negative predictive value (NPV) for a prediction made on the first day of hospitalization and for the maximum prediction made over the entire admission. One-sided McNemar's tests were used when comparing the sensitivity and specificity of the model against that of the M-MST. The null hypothesis was that the M-MST performance was equal to or better than that of the model, at a 0.025 significance threshold.

We also generated a receiver operating characteristic curve (ROC) and a precision–recall curve (PRC) and determined areas under them (AUROC and AUPRC, respectively) to assess model ranking ability. 38 While both the AUROC and AUPRC are important to assess model ranking ability, the AUPRC may offer additional insight with class imbalance. 39 40 For AUROC and AUPRC analyses, 95% confidence intervals (CIs) were generated with a bootstrap method. 41 42 We employed early stopping criteria such that our validation set was used to track the performance of model accuracy as we trained the model. After 100 iterations without improvement, we rolled the model back to the checkpoint with the highest AUROC.

Additional analyses calculated patients' length of stay and Charlson Comorbidity Index, as well as 30-, 60-, and 90-day hospital mortality and readmission rates. 43 The Kaplan–Meier estimator generated readmission and mortality rates. 44 45 To explain relative feature importance in model risk scores for individual observations, we examined Shapley Additive Explanations (SHAP) values and created waterfall plots. We explored model fairness and how well the model was calibrated by visually inspecting bins of actual versus expected cases within deciles segmented by race and sex. Finally, we inspected when the model flagged patients at risk for malnutrition in relation to when it was documented by a dietitian, as well as the agreement between dietitian-recorded malnutrition and discharge-coded malnutrition.

All analyses were run in Python 3.10. The study was approved by the Cedars-Sinai Medical Center Institutional Review Board.

## Results

Among 166,841 hospital admissions, including 106,449 unique patients, during a period of nearly 3.75 years, 7.6% carried a diagnosis of malnutrition of any type by discharge coding. Baseline characteristics of the admissions are shown in Table 1 and Supplementary Table S5 [available in the online version only]). Significant bivariate differences existed between those who were coded as malnourished and those who were not, using Fisher's exact test or Welch's *t* -test. Patients who were malnourished were generally older, had a longer length of hospital stay, were admitted to the intensive care unit (ICU) more often, and were more frequently covered by Medicare. Admitting diagnoses were also significantly different between the two groups ( *p* < 0.0001): Those who were malnourished were more likely to carry a diagnosis on admission of urinary tract infection, shortness of breath, anemia, kidney failure, sepsis, or pneumonia; by contrast, chest pain was significantly more common in the non-malnourished group compared with the malnourished group.

**Table 1:** Baseline characteristics of hospital admissions

	Total ( *n* = 106,449)	Malnourished ( *n* = 7,273)	Not malnourished ( *n* = 99,176)	*p* -Value
Age (y)				
Median (IQR)	60 (38–74)	72 (59–83)	58 (38–73)	*p* < 0.0001
Gender ( *n* , %)				
Female Male	61,511 (57.8%) 44,929 (42.2%)	3,454 (47.5%) 3,819 (52.5%)	58,057 (58.5%) 41,110 (41.5%)	*p* < 0.0001 *p* < 0.0001
Ethnicity ( *n* , %)				
Asian Black African American Hispanic White Other/Unknown	8,307 (7.8%) 14,769 (13.9%) 17,205 (16.2%) 59,313 (55.7%) 6,849 (6.4%)	629 (8.6%) 1,178 (16.2%) 1,108 (15.2%) 3,794 (52.2%) 564 (7.8%)	7,678 (7.7%) 13,591 (13.7%) 16,097 (16.2%) 55,519 (56.0%) 6,285 (6.3%)	*p* = 0.0061 *p* < 0.0001 *p* = 0.0259 *p* < 0.0001 *p* < 0.0001
Primary diagnosis upon admission (number of encounters, percentage of all encounters)				
Urinary tract infection Shortness of breath Chest pain Acute kidney failure Anemia COVID-19 Sepsis Weakness Pneumonia Hypoosmolality and hyponatremia Other	3,684 (3.5%) 3,575 (3.4%) 2,597 (2.4%) 2,408 (2.3%) 2,008 (1.9%) 1,899 (1.8%) 1,703 (1.6%) 1,681 (1.6%) 1,335 (1.3%) 1,231 (1.2%) 84,328 (79.2%)	616 (8.5%) 381 (5.2%) 101 (1.4%) 387 (5.3%) 303 (4.2%) 151 (2.1%) 358 (4.9%) 281 (3.9%) 205 (2.8%) 219 (3.0%) 4,271 (58.7%)	3,068 (3.1%) 3,194 (3.2%) 2,496 (2.5%) 2,021 (2.0%) 1,705 (1.7%) 1,748 (1.8%) 1,345 (1.4%) 1,400 (1.4%) 1,130 (1.1%) 1,012 (1.0%) 80,057 (80.7%)	*p* < 0.0001 *p* < 0.0001 *p* < 0.0001 *p* < 0.0001 *p* < 0.0001 *p* < 0.0001 *p* < 0.0001 *p* = 0.0073 *p* < 0.0001 *p* < 0.0001 *p* < 0.0001
Length of stay (d), median (IQR)	3.4 (2.2–6.1)	10.1 (5.6–18.9)	3.2 (2.2–5.5)	*p* < 0.0001
Level of care (number of admissions)				
Intensive care unit Non-intensive care unit	4,195 (3.9%) 102,254 (96.1%)	836 (11.5%) 6,437 (88.5%)	3,359 (3.4%) 95,817 (96.6%)	*p* < 0.0001 *p* < 0.0001
Payer ( *n* , %)				
Private Medicare Self-pay Medi-Cal	57,197 (53.7%) 36,950 (34.7%) 7,137 (6.7%) 5,165 (4.9%)	2,692 (37.0%) 3,866 (53.2%) 345 (4.7%) 370 (5.1%)	54,505 (55.0%) 33,084 (33.4%) 6,792 (6.8%) 4,795 (4.8%)	*p* < 0.0001 *p* < 0.0001 *p* < 0.0001 *p* = 0.3364

Notes: Malnourished patients are those who received a discharge code of malnutrition.

*p* -Values for continuous variables were determined with bootstrap analysis (sample size 9,999). For categorical variables, Fisher's exact test was used for all measures, except for age and length of stay, which were determined by Welch's *t* -test.

Model performance metrics are shown in Table 2 . The model outperformed the nurse-administered M-MST when comparing sensitivity and specificity in non-ICU units, and overall, with coded malnutrition or dietitian notes as reference (also see Supplementary Table S6 [available in the online version only]). For example, when dietitian-recorded malnutrition was the reference standard, the model was 49% more sensitive overall than the M-MST in identifying cases (0.52 [95% CI: 0.48–0.56] vs. 0.35 [95% CI: 0.31–0.39]) on the first day of hospitalization. When coded malnutrition at discharge was the reference, the model was 204% more sensitive overall in identifying cases (0.49 [95% CI: 0.46–0.52] vs. 0.24 [95% CI: 0.21–0.26]) on the first day of hospitalization. The model generally had higher AUROC, sensitivity, and negative predictive values for non-ICU admissions compared with ICU admissions, using both coded and recorded malnutrition as references. Model performance metrics were generally the same or higher when using the highest predicted risk throughout hospitalization compared with only using the prediction generated on the first day. Null hypotheses were rejected.

**Table 2:** Predictive model performance metrics (95% confidence interval)

	Discharge-coded malnutrition			Dietitian-recorded malnutrition		
Hospital location	Intensive care unit	Non-intensive care units	Overall	Intensive care unit	Non-intensive care units	Overall
Number of admissions flagged (number of discharge coded or dietitian recorded cases of malnutrition)	519 (116)	13,575 (955)	14,094 (1,071)	519 (42)	13,575 (628)	14,094 (670)
Malnutrition rate, discharge-coded or dietitian-recorded	22.40%	7.00%	7.60%	8.10%	4.60%	4.80%
Risk of malnutrition on the first day of hospitalization						
Model risk threshold	65.1%	39.7%	42.2%	65.1%	39.7%	42.2%
Admissions identified ( *n* , percentage of all admissions)	47 (9.1%)	903 (6.7%)	950 (6.7%)	47 (9.1%)	903 (6.7%)	950 (6.7%)
AUROC	0.81 (0.76–0.85)	0.92 (0.91–0.93)	0.92 (0.91–0.92)	0.80 (0.71–0.85)	0.92 (0.91–0.92)	0.91 (0.90–0.92)
AUPRC	0.58 (0.47–0.66)	0.55 (0.52–0.58)	0.55 (0.52–0.58)	0.22 (0.14–0.31)	0.42 (0.38–0.46)	0.40 (0.36–0.44)
Sensitivity	0.28 (0.21–0.37)	0.50 (0.47–0.53)	0.49 (0.46–0.52)	0.26 (0.14–0.41)	0.53 (0.49–0.57)	0.52 (0.48–0.56)
Specificity	0.97 (0.94–0.98)	0.97 (0.96–0.97)	0.97 (0.96–0.97)	0.92 (0.90–0.95)	0.96 (0.95–0.96)	0.96 (0.95–0.96)
Positive predictive value	0.70 (0.56–0.82)	0.53 (0.50–0.56)	0.55 (0.52–0.58)	0.23 (0.13–0.38)	0.37 (0.34–0.40)	0.37 (0.34–0.40)
Negative predictive value	0.82 (0.79–0.86)	0.96 (0.96–0.97)	0.96 (0.95–0.96)	0.93 (0.91–0.95)	0.98 (0.97–0.98)	0.98 (0.97–0.98)
Maximum risk of malnutrition during hospitalization						
Model risk threshold	85.50%	65.40%	68.10%	85.50%	65.40%	68.10%
Admissions identified ( *n* , percentage of all admissions)	47 (9.1%)	903 (6.7%)	950 (6.7%)	47 (9.1%)	903 (6.7%)	950 (6.7%)
AUROC	0.91 (0.88–0.93)	0.95 (0.95–0.96)	0.95 (0.95–0.96)	0.83 (0.78–0.87)	0.94 (0.93–0.94)	0.93 (0.92–0.94)
AUPRC	0.76 (0.68–0.83)	0.66 (0.63–0.69)	0.67 (0.64–0.70)	0.22 (0.14–0.30)	0.46 (0.42–0.50)	0.43 (0.39–0.47)
Sensitivity	0.33 (0.25–0.42)	0.59 (0.56–0.63)	0.58 (0.55–0.61)	0.17 (0.07–0.31)	0.58 (0.54–0.62)	0.56 (0.52–0.60)
Specificity	0.98 (0.96–0.99)	0.97 (0.97–0.98)	0.97 (0.97–0.98)	0.92 (0.89–0.94)	0.96 (0.96–0.96)	0.96 (0.95–0.96)
Positive predictive value	0.81 (0.67–0.91)	0.63 (0.60–0.66)	0.66 (0.63–0.69)	0.15 (0.06–0.28)	0.40 (0.37–0.43)	0.39 (0.36–0.43)
Negative predictive value	0.83 (0.80–0.87)	0.97 (0.97–0.97)	0.97 (0.96–0.97)	0.93 (0.90–0.95)	0.98 (0.98–0.98)	0.98 (0.98–0.98)
Risk of malnutrition per nurse administered modified malnutrition screening tool (M-MST)						
Admissions identified ( *n* , percentage of all admissions)	47 (9.1%)	903 (6.7%)	950 (6.7%)	47 (9.1%)	903 (6.7%)	950 (6.7%)
AUROC	NA	NA	NA	NA	NA	NA
AUPRC	NA	NA	NA	NA	NA	NA
Sensitivity	0.22 (0.15–0.31)	0.24 (0.21–0.26)	0.24 (0.21–0.26)	0.40 (0.26–0.56)	0.35 (0.31–0.38)	0.35 (0.31–0.39)
Specificity	0.95 (0.92–0.97)	0.95 (0.94–0.95)	0.95 (0.94–0.95)	0.94 (0.91–0.96)	0.95 (0.94–0.95)	0.95 (0.94–0.95)
Positive predictive value	0.55 (0.41–0.69)	0.25 (0.22–0.28)	0.27 (0.24–0.29)	0.36 (0.23–0.51)	0.24 (0.21–0.27)	0.25 (0.22–0.27)
Negative predictive value	0.81 (0.77–0.84)	0.94 (0.94–0.95)	0.94 (0.93–0.94)	0.95 (0.92–0.96)	0.97 (0.96–0.97)	0.97 (0.96–0.97)

Abbreviations: AUPRC, area under the precision–recall curve; AUROC, area under the receiver operating characteristic curve; M-MST, nurse-administered, modified version of the Malnutrition Screening Tool.

Notes: Dietitian record of malnutrition by ASPEN and AND Indicators for Malnutrition (AAIM) criteria.

For comparisons between the model and the M-MST, the model's risk threshold was adjusted such that it flagged the same number of patients at risk of malnutrition as the M-MST.

Confidence intervals for AUROC, AUPRC, sensitivity, specificity, positive predictive value, and negative predictive value were generated by bias-corrected and accelerated bootstrap (BCa method) with 95% confidence intervals (sample size 9,999).

**Fig. 2:**
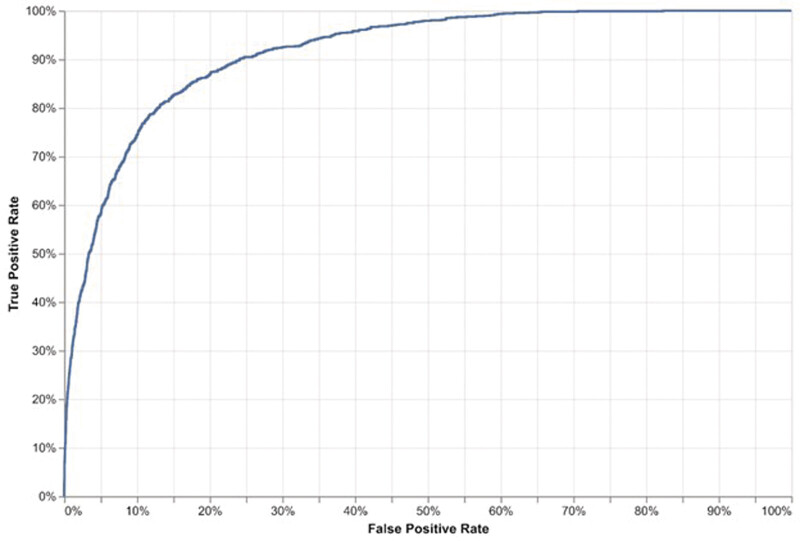
Receiver operating characteristic curve, first day of hospitalization. Area under the receiving operating characteristic curve (AUROC) 91.5%.

**Fig. 3:**
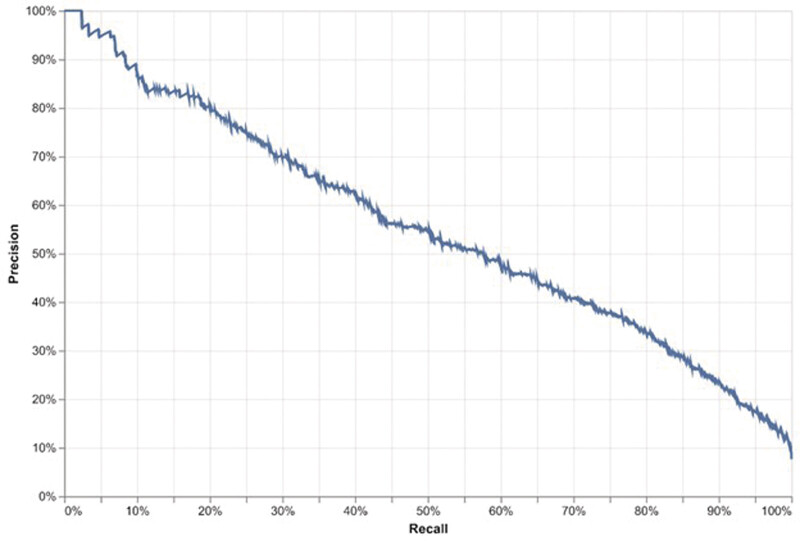
Precision–recall curve, first day of hospitalization. Area under the precision–recall curve (AUPRC) 55.4%, malnutrition rate 7.6%.

Against the reference standard of coded malnutrition, the model's AUROC was 0.92 (95% CI: 0.91–0.92) on the first day of hospitalization and rose to 0.95 (95% CI: 0.95–0.96) using the highest predicted risk throughout a patient's hospitalization ( Table 2 ; Fig. 2 and Supplementary Fig. S2 [available in the online version only]). AUPRC values rose in a similar fashion, although to a greater extent ( Table 2 ; Fig. 3 and Supplementary Fig. S3 [available in the online version only]). AUROC values overall were similar when the reference standard was either coded malnutrition or dietitian-recorded malnutrition, while AUPRC values were higher with coded malnutrition ( Table 2 ). OOT sampling demonstrated that the trends captured by the model held over time (validation set first day of hospitalization AUROC 91.77% and AUPRC 53.52%, with base malnutrition rate of 7.03%; data not shown).

By comparison to patients flagged by the M-MST, patients identified by the model were significantly sicker, as reflected in the higher degree of comorbidity (Charlson Comorbidity Index 6.4 vs. 5.3; *p* < 0.0001) and required a significantly longer length of stay (13.4 vs. 9.5 days; *p* < 0.0001), using Fisher's exact test or Welch's *t* -test ( Table 3 ). Patients identified by the model had higher readmissions and mortality over 30, 60, 90 days as determined by the Kaplan–Meier estimator, when compared with those identified by the M-MST ( Table 3 and Supplementary Table S6 [available in the online version only]). Few patients who were neither flagged by the model nor the M-MST died during the follow-up period ( Table 3 ).

**Table 3:** Hospital mortality and readmission rates predicted by model and modified Malnutrition Screening Tool (95% confidence interval)

	Model on the first day of hospitalization		Nurse-administered screening tool (M-MST)		Absolute difference (flagged)	Relative difference (flagged)	Difference *p* -value (flagged) ^a^
	Not flagged	Flagged	Not flagged	Flagged			
Number of admissions	13,143	951	13,144	950			
Length of stay	6.1 (5.9–6.3)	13.4 (12.4–15.0)	6.4 (6.2–6.6)	9.5 (8.8–10.3)	3.9	41.00%	*p* < 0.0001
Charlson's Comorbidity Index	3.0 (3.0–3.1)	6.4 (6.2–6.6)	3.1 (3.1–3.2)	5.3 (5.1–5.5)	1.1	21.50%	*p* < 0.0001
Readmission							
30-day	0.05 (0.05–0.06)	0.12 (0.10–0.14)	0.06 (0.05–0.06)	0.09 (0.07–0.11)	2.60%	29.30%	*p* = 0.0414
60-day	0.08 (0.07–0.08)	0.17 (0.14–0.20)	0.08 (0.07–0.08)	0.14 (0.11–0.16)	2.90%	21.50%	*p* = 0.0698
90-day	0.09 (0.08–0.10)	0.20 (0.17–0.24)	0.09 (0.09–0.10)	0.15 (0.13–0.18)	5.30%	35.10%	*p* = 0.0039
Mortality							
30-day	0.02 (0.02–0.02)	0.17 (0.14–0.19)	0.03 (0.02–0.03)	0.09 (0.07–0.11)	7.50%	85.30%	*p* < 0.0001
60-day	0.03 (0.02–0.03)	0.22 (0.20–0.25)	0.03 (0.03–0.04)	0.12 (0.10–0.15)	9.90%	81.10%	*p* < 0.0001
90-day	0.03 (0.03–0.03)	0.25 (0.22–0.29)	0.04 (0.03–0.04)	0.15 (0.12–0.17)	10.70%	72.80%	*p* < 0.0001

Abbreviation: M-MST, nurse-administered, modified version of the Malnutrition Screening Tool.

Notes: Statistical significance tests were performed on only the non-overlapping observations (those flagged by both the model and M-MST were excluded).

The model had 711 observations, and the M-MST had 710 observations.

The model risk threshold was 41.5%.

Kaplan–Meier estimator was used to estimate readmission and mortality rates for those flagged by the model and for those flagged by M-MST.

*p* -Values for differences in readmissions and mortality via the log(−log(·)) using *lifelines* Python package version 0.29.0.

Confidence intervals for mortality and readmission rates were generated by the Greenwood exponential formula with 95% confidence intervals.

*p* -Values for differences in length of stay and Charlson Comorbidity Indices were generated by Welch's *t* -test and others by Fisher's exact test.

Confidence intervals for length of stay and Charlson Comorbidity Index generated by bias-corrected and accelerated bootstrap (BCa method) with 95% confidence intervals (sample size 9,999).

Feature importance for two individual admissions of varying malnutrition risk is shown in Supplementary Figs. S4 and S5 (available in the online version only). Albumin and BMI were the top two features of importance. We did not observe evidence of model unfairness, nor substantial model over- or underpredicting, for groups stratified by race or sex ( Supplementary Figs. 6–12 [available in the online version only]).

In comparison to the first time that a dietitian documented malnutrition in the EHR, the model flagged malnutrition earlier 72.7% of the time, later 10.4% of the time, and did not flag a patient 16.9% (data not shown). Overall, the model offered a median advance identification of 1.3 days (with a mean of 4.0 days; data not shown). There was high agreement between the presence or absence of dietitian-recorded and discharge-coded malnutrition ( Supplementary Table S7 [available in the online version only]).

## Discussion

In this large retrospective analysis, we found that a novel AI model identified patients at risk of malnutrition with a high degree of sensitivity and specificity overall when indexed against either dietitian-recorded malnutrition or discharge-coded malnutrition. The model demonstrated greater performance metrics than the version of the MST that was used in practice, as well as a greater ability to identify patients at risk for death and readmission. While model performance improved by using data captured throughout hospitalization, a significant benefit over the existing tool was observed on the first day alone.

This research is part of a long history of MST development and evaluation. 11 12 Our study found improved performance over a modified version of the MST, the instrument recommended by the Academy of Nutrition and Dietetics for malnutrition screening. 14 The MST is reported to have moderate performance metrics (sensitivity, specificity, NPV, PPV between 80 and ≤89%). 14 Our model's AUROC, which integrates sensitivity and specificity, was observed to be over 90% overall, indexed against dietitian-coded malnutrition and discharge-coded malnutrition, on both the first day of hospitalization and throughout hospitalization.

Recently, investigators have begun developing screening tools with machine learning models. One reported an overall sensitivity of 0.35 (95% CI: 0.33, 0.37) and specificity of 0.93 (95% CI: 0.92, 0.94) following calibration, 46 47 while another reported an AUROC of 0.85 to 0.87 and AUPRC of 0.19 to 0.26 (with malnutrition prevalence ranging from 4.0 to 6.2%). 48 Our overall performance metrics, with dietitian notes or hospital codes as reference, appear higher than these, and may be due to a broader range of features (e.g., flow sheet data with percentage of a meal eaten) and different features engineering (e.g., trends in BMI ranging from 3 to 365 days). While there are no thresholds set for AUPRC, an AUROC of 80 to 90% is considered “excellent,” and more than 90%, which we achieved, is considered “outstanding.” 49

We observed an overall coded malnutrition rate of 7.6%, which is lower than the malnutrition rate reported in the global literature using clinical tools such as the Subjective Global Assessment, but is aligned with the 8.9% rate reported in the United States based on hospital coding. 5 50 Of note, albumin, a notable feature in our SHAP value analysis, is not considered a definitive marker of malnutrition due to its lack of responsiveness to acute changes in nutrient intake. 51 However, its specificity may be enhanced when considered in conjunction with other biomarkers and clinical indicators, and as such, its prominence among the SHAP values can support its inclusion as part of a broad, multifactorial assessment framework.

To the best of our knowledge, using LLMs in the manner we did for extracting data from clinician notes to identify hospital malnutrition risk has not been done before. One prior study applied an LLM with retrieval augmented generation to summarize and extract clinical information about malnutrition from EHRs in aged care facilities. 52 Another employed natural language processing, also in aged care facilities, to assess for malnutrition from notes written about residents. 53 In that study, the structured, tabulated data recorded 48% of malnourished clients, while 82% were identified from the progress notes. By comparison to these studies, our incorporation of a supervised machine learning model with the LLM offered the potential to uncover previously unknown patterns, such as complex interactions among laboratory values or subtle temporal trends in patient data, which may be predictive of malnutrition. Future efforts may be able to extract more value from an LLM, including the querying of more notes with more questions, or the use of named entity recognition to create new datasets.

Our study has notable strengths. The ethical use of our AI model was approved by the medical center's Institutional Review Board. The large EHR dataset included admissions with many different admitting diagnoses and varying levels of acuity. To compare the model's performance against other screening tools at the time that such tools are expected to be applied, the model was evaluated on the first day of hospitalization. To avoid spurious findings, as well as overfitting, the data were split between training, validation, and test sets such that the model was trained on the training set and the hyperparameters were tuned on results from the validation set but not from the test set. 54

While it may not be surprising that an AI approach using a large volume of clinical data would perform better than a two-question M-MST screening tool, we evaluated the model against dietitian-recorded malnutrition and coded malnutrition (criterion validity), as well as its ability to identify future mortality and hospital readmissions (predictive validity). We observed stability of model performance when comparing the validation and test sets, suggesting the model is stable for these time horizons. OOT sampling also helped ensure model reliability for future predictions. Additional work may be done to determine whether the model retains performance over longer time horizons and to monitor the model for performance drift that could indicate the need for refitting or retraining on more recent data. Importantly, we found that there were no instances of substantial model over- or underpredicting for groups stratified by race or sex.

Study limitations include that the dataset was from a single hospital using inpatient data. Yet with the breadth of demographic and health characteristics of patients admitted, the results likely have strong external generalizability. Importantly, too, the features selected by the model are widely available at other hospitals. The study was retrospective in nature and future research may look at the model's performance in a prospective fashion. We recognize that the minor modification of the MST as used by nurses means that it is different than the validated version of the tool. We also acknowledge that correlations may exist between some of the features, and these may affect the interpretation of SHAP values. Discharge codes may be susceptible to undercoding or misclassification, while dietitian notes may vary in consistency or availability. Bias may exist in the diagnosis of malnutrition (for instance, certain clinicians may be more likely to make the diagnosis than others); however, we aimed to mitigate this risk in part by using clinician notes and observations of nutrition status and appetite and by not including a patient's unit (where certain clinicians may regularly work) as a model feature. It is possible that there may be distinct clinical or institutional practices at other health care sites, such as with diagnosis and coding, and a next step may include evaluating model performance at additional clinical sites.

However, altogether, the study presents important implications for patients, clinicians, and health care delivery systems. For patients, the time to generate a malnutrition risk score currently takes less than 1 hour, including data transfer, processing, inference, and making predictions available, thus allowing the model to screen every inpatient every day. Earlier identification of malnutrition risk may lead to earlier in-depth assessment, diagnosis, and nutritional care for ill patients, resulting in better health, lower mortality, shorter hospital stays, and fewer readmissions. 8 9 55 56 57 For clinicians, the model may potentially be paired with clinical documentation assistance or additional decision support systems. 58 59 For health care delivery systems, operational efficiencies may be gained as the model enables passive and continuous screening with EHR data, as opposed to current screening methods, which often require administration, scoring, and interpretation of questionnaires. Such efficiencies can free up clinician time for other patient care activities. The model also affords evaluation of massive amounts of data and recognition of potentially previously unforeseen patterns in short periods of time. By improving the prediction of health care service utilization, such as readmissions, payers and providers may make more accurate financial projections.

## Conclusion

In conclusion, the screening, diagnosis, and timely management of malnutrition in hospitals remains a significant challenge. AI, with its ability to analyze massive amounts of data quickly and continuously, when applied ethically, provides a promising applied clinical informatics solution, and one that can be coupled with clinician-delivered patient care. The model described herein has predictive capabilities of timely and measurable importance to patients, providers, and payers of health care.

## Clinical Relevance Statement

Study findings provide validation for a novel machine learning model's use in the prediction of in-hospital malnutrition, a condition that remains underdiagnosed and undertreated and yet may have severe or fatal consequences.

## Multiple-Choice Questions

1. What were the two types of validity presented in this study, and why are these of importance?
  - Face validity and criterion validity
  - Criterion validity and predictive validity
  - Predictive validity and construct validity
  - Construct validity and face validity
  **Correct Answer** : The correct answer is option b. While face validity (does the test make sense at face value?) and construct validity (does the test align with theoretical constructs?) may also be of value when developing new screening or diagnostic tests, the research herein evaluated criterion validity (does the test align with certain clinical criteria?) as well as predictive validity (does the test have meaningful predictive abilities?). By examining coded malnutrition and dietitian-recorded malnutrition, as well as future mortality and hospital readmissions, the research presents validity of clinically meaningful importance.
2. Large language models may contribute to malnutrition screening in many ways. Which was one way explored in this study?
  - Assisting with the manual review of electronic health record data.
  - Eliminating the need for electronic health records.
  - Extracting relevant information from unstructured data in clinical notes to generate predictive model features.
  - Generating clinical documentation.
  **Correct Answer** : The correct answer is option c. Large language models allow for automated, rather than manual, review of data and have leveraged, rather than replaced, electronic health records. While these models may assist with generating clinical documents, in the research presented here, they were employed to read through clinical documents, answer relevant questions, and generate model features. In this way, the predictive model leveraged both structured and unstructured health record data.
