# Supplementary material for: Artificial Intelligence-Based Hospital Malnutrition Screening: Validation of a Novel Machine Learning Model
Source: Appl Clin Inform. 2025 Nov 14;16(5):1646–57. doi: 10.1055/a-2635-3158 (PMC12618146; doi:10.1055/a-2635-3158)
Supplement: Supplementary file 1 — Supplementary Material [file 10-1055-a-2635-3158_26849736.pdf]

# Supplementary Appendix

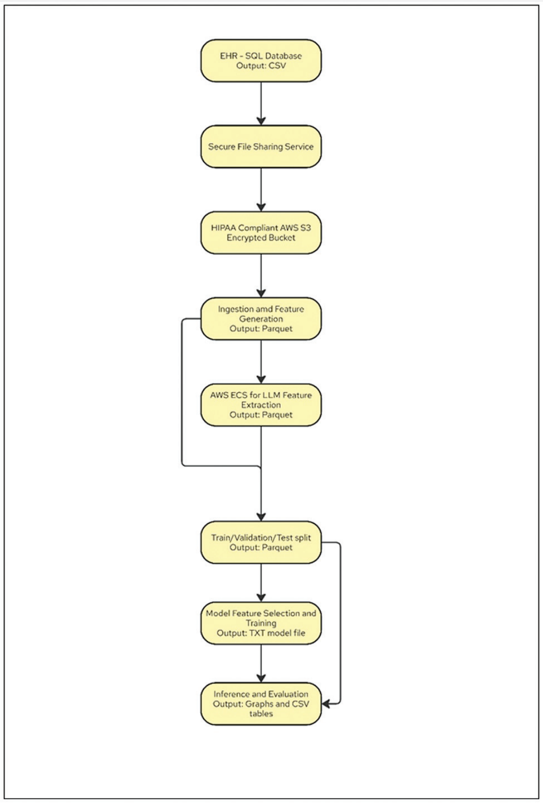


**Supplementary Fig. S1** Data flow. AWS, Amazon Web Services; CSV, comma-separated value; ECS, Elastic Container Service; EHR, electronic health record; HIPAA, Health Insurance Portability and Accountability Act; LLM, large language model; SQL, structured query language; TXT, text.

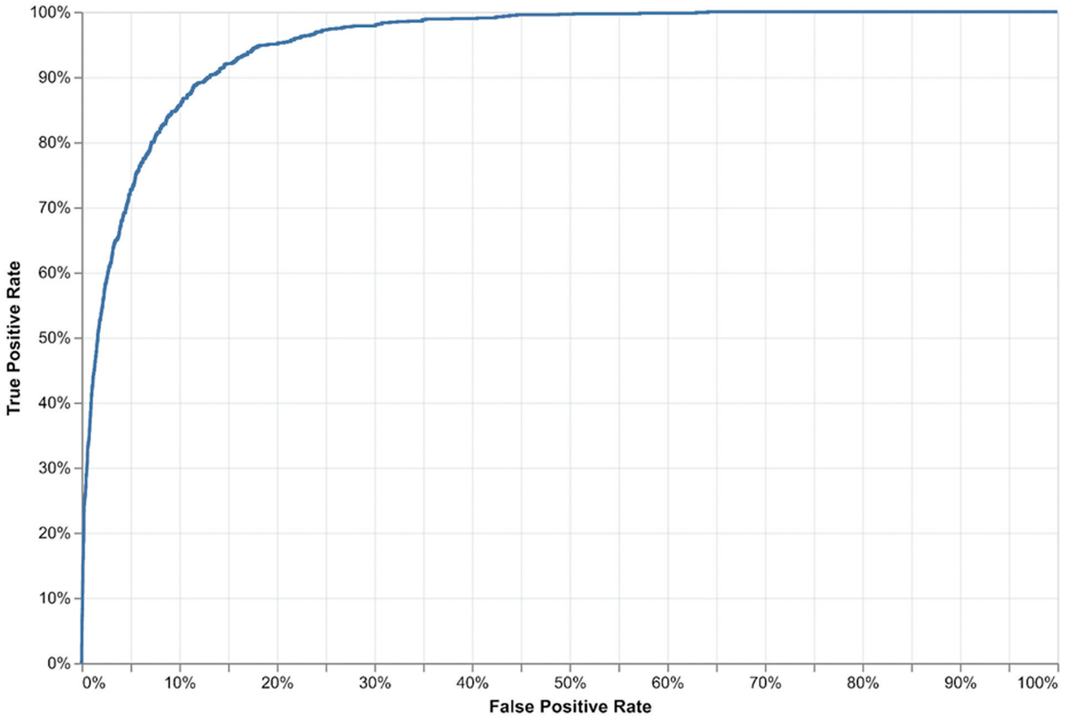


**Supplementary Fig. S2** Receiver operating characteristic curve, maximum risk during hospitalization (AUROC 95.3%). AUROC, area under the receiver operating characteristic curve.

 © 2025. The Author(s).

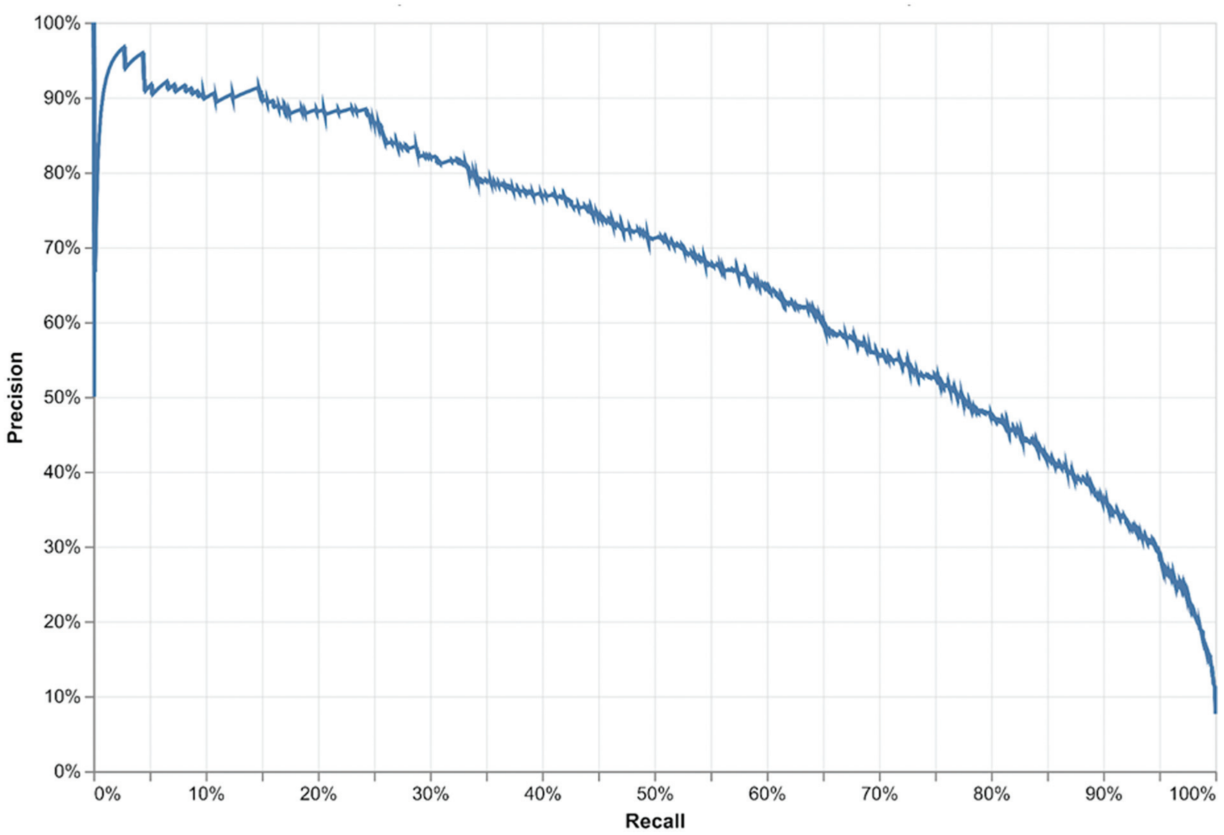


**Supplementary Fig. S3** Precision–recall curve, maximum risk during hospitalization (AUPRC 67.0%; malnutrition rate 7.6%). AUPRC, area under the precision–recall curve.

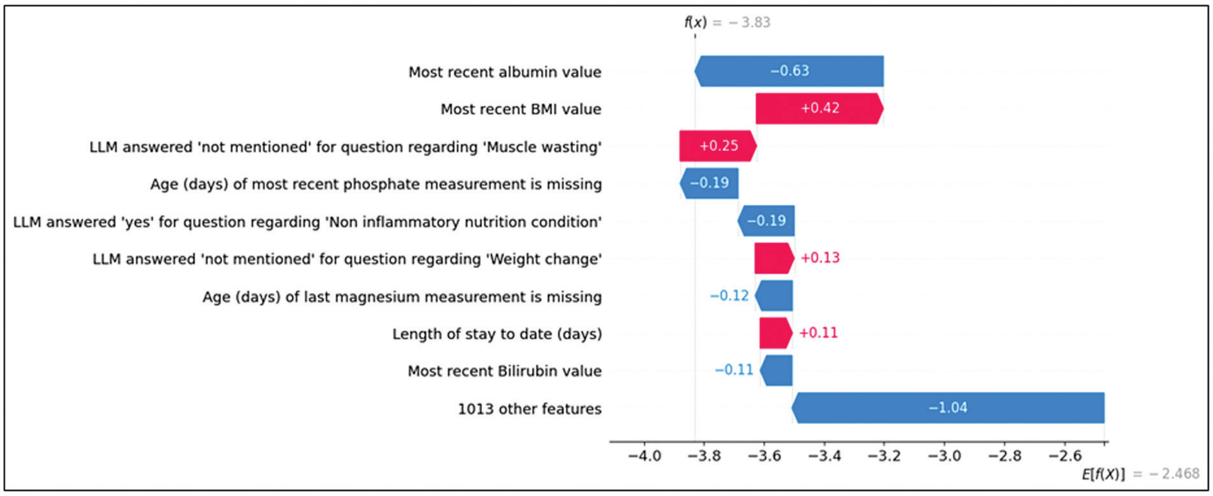


**Supplementary Fig. S4** Feature importance for patient at low risk of malnutrition. BMI, body mass index; Ef(X), logit for the population; f(x), logit for the individual; LLM, large language model.

 © 2025. The Author(s).

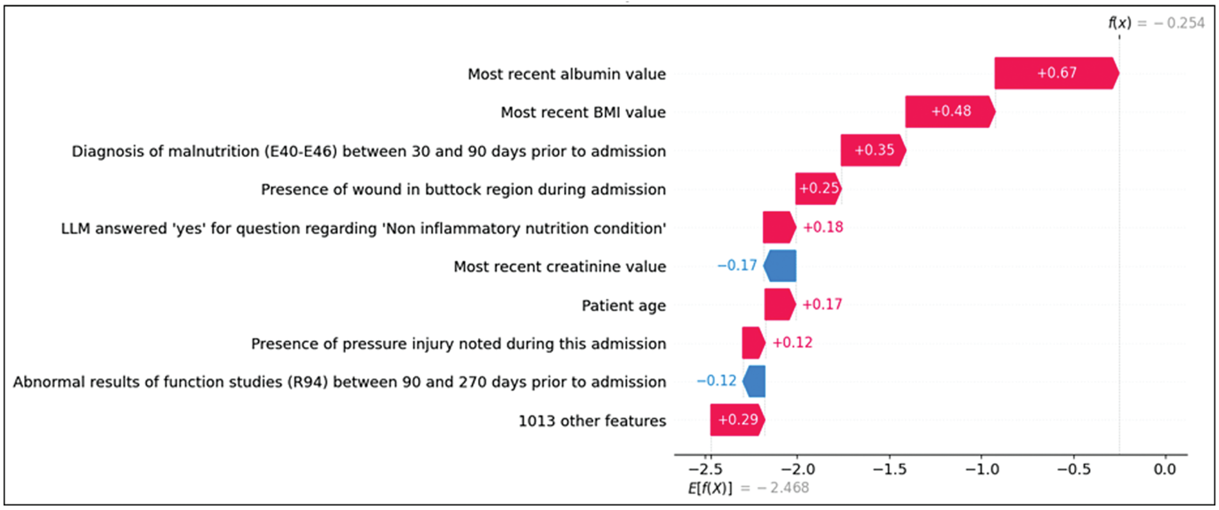


**Supplementary Fig. S5** Feature importance for patient at high risk of malnutrition. BMI, body mass index; Ef(X): logit for the population; f(x): logit for the individual; LLM, large language model.

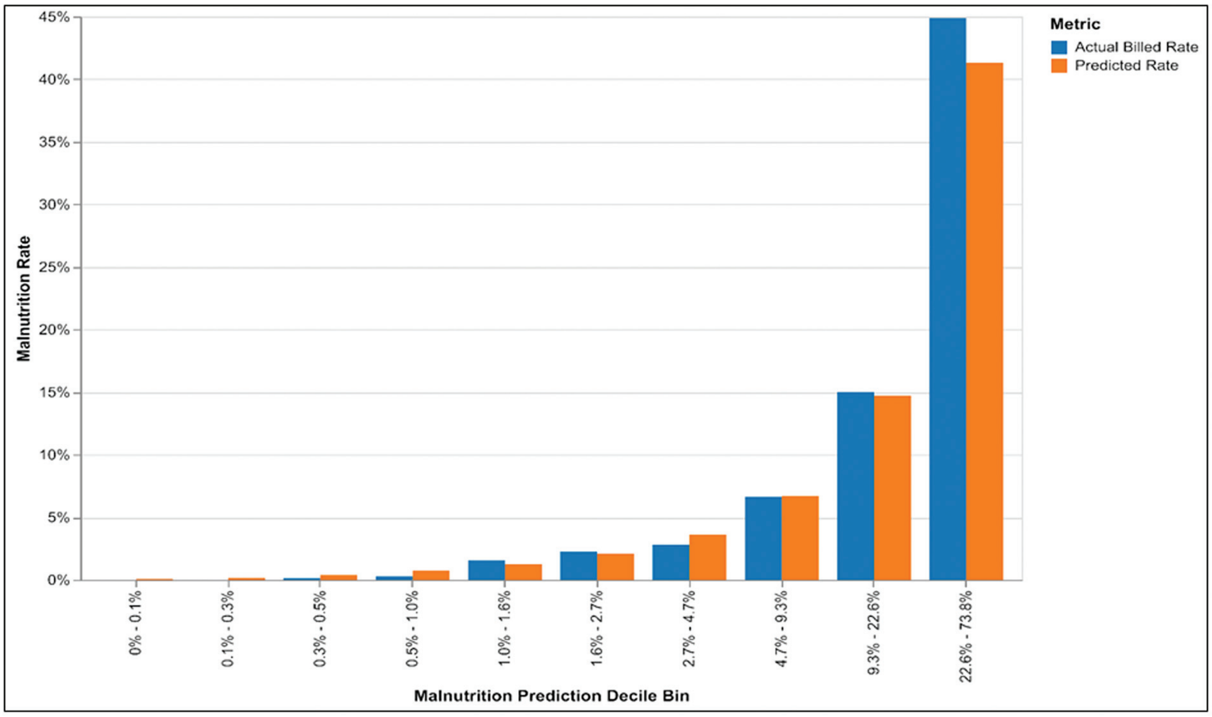


**Supplementary Fig. S6** Actual versus predicted rate of malnutrition for patients who reported sex as female. Test set with 8,098 female patients; actual billed rate, rate of coded malnutrition within each decile; predicted rate, mean model risk score within each decile.

 © 2025. The Author(s).

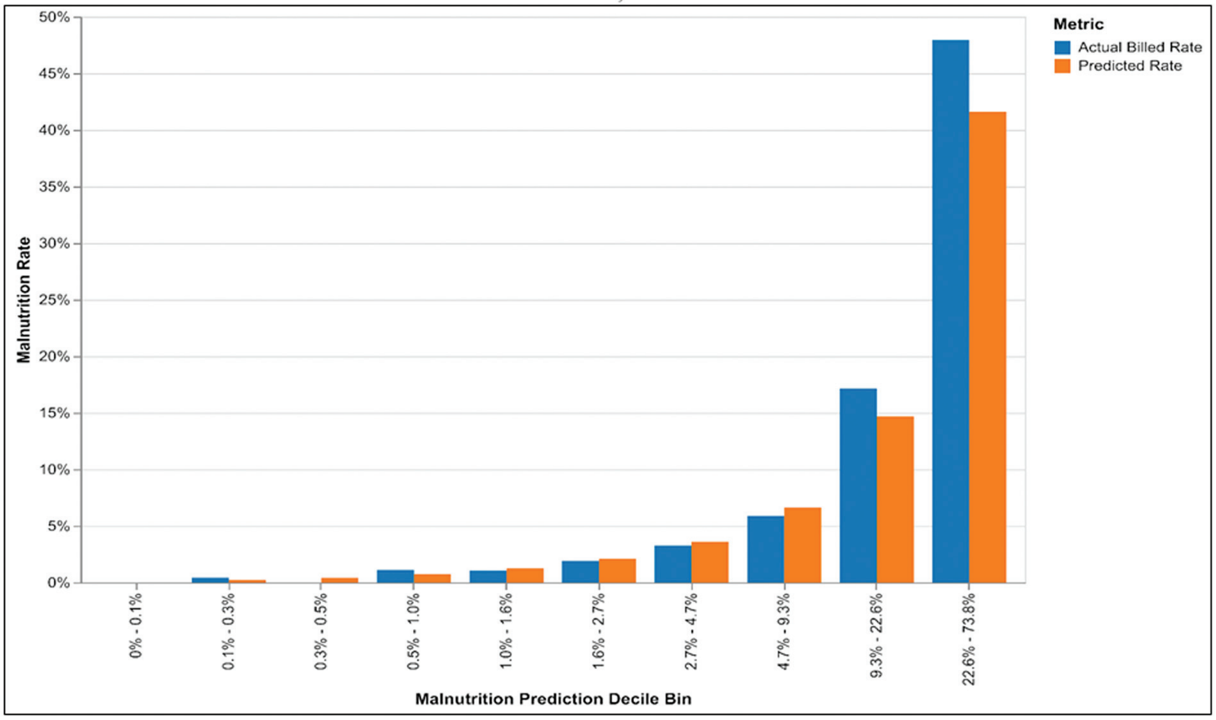


**Supplementary Fig. S7** Actual versus predicted rate of malnutrition for patients who reported sex as male. Test set with 5,996 male patients; actual billed rate, rate of coded malnutrition within each decile; predicted rate, mean model risk score within each decile.

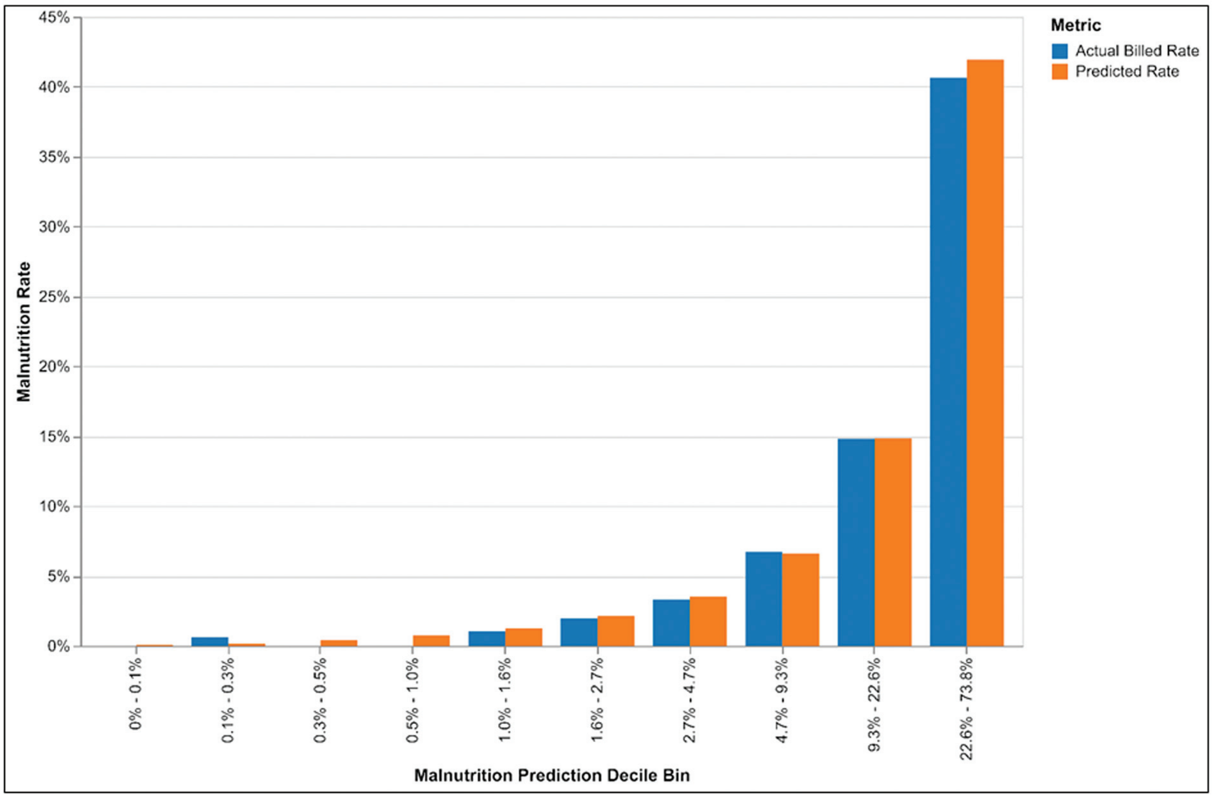


**Supplementary Fig. S8** Actual versus predicted rate of malnutrition patients who reported race as Asian. Test set with 1,149 patients who reported race as Asian; actual billed rate, rate of coded malnutrition within each decile; predicted rate, mean model risk score within each decile.

 © 2025. The Author(s).

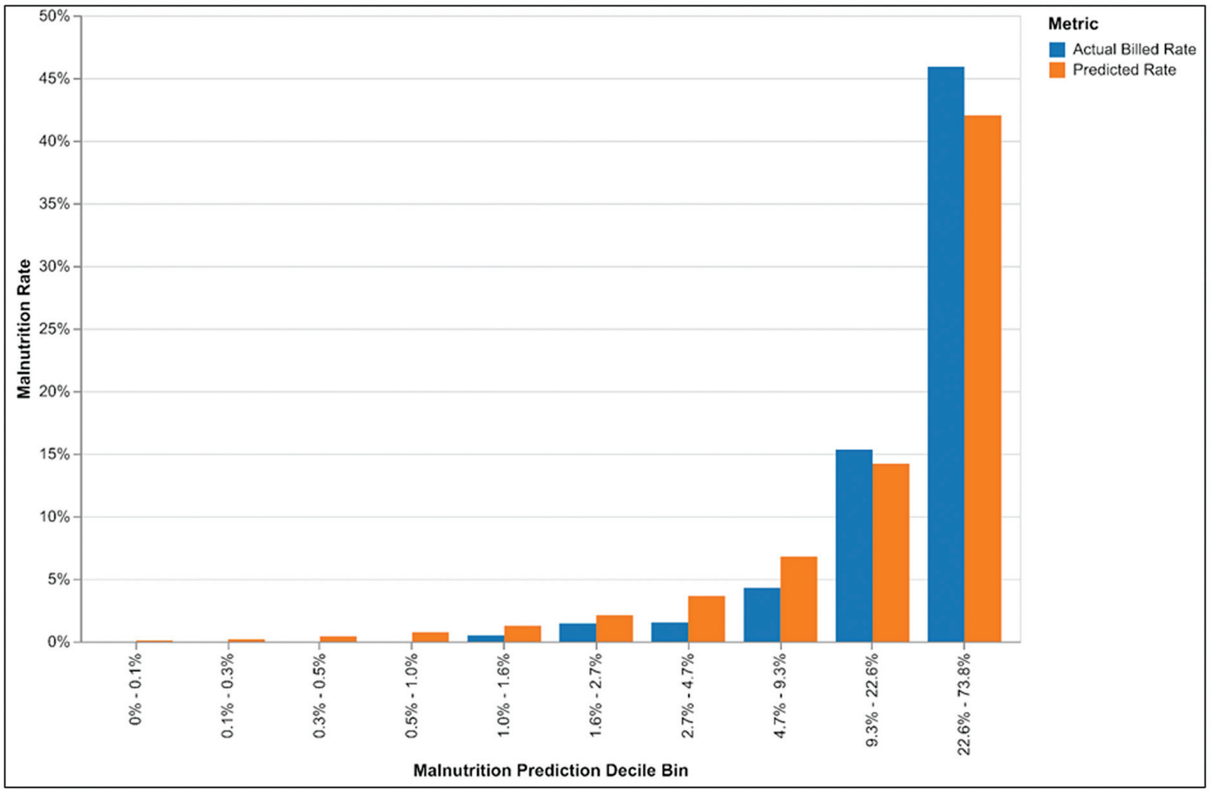


**Supplementary Fig. S9** Actual versus predicted rate of malnutrition patients who reported race as Black or African American. Test set with 1,929 patients who reported race as Black or African-American; Actual billed rate, rate of coded malnutrition within each decile; Predicted rate, mean model risk score within each decile.

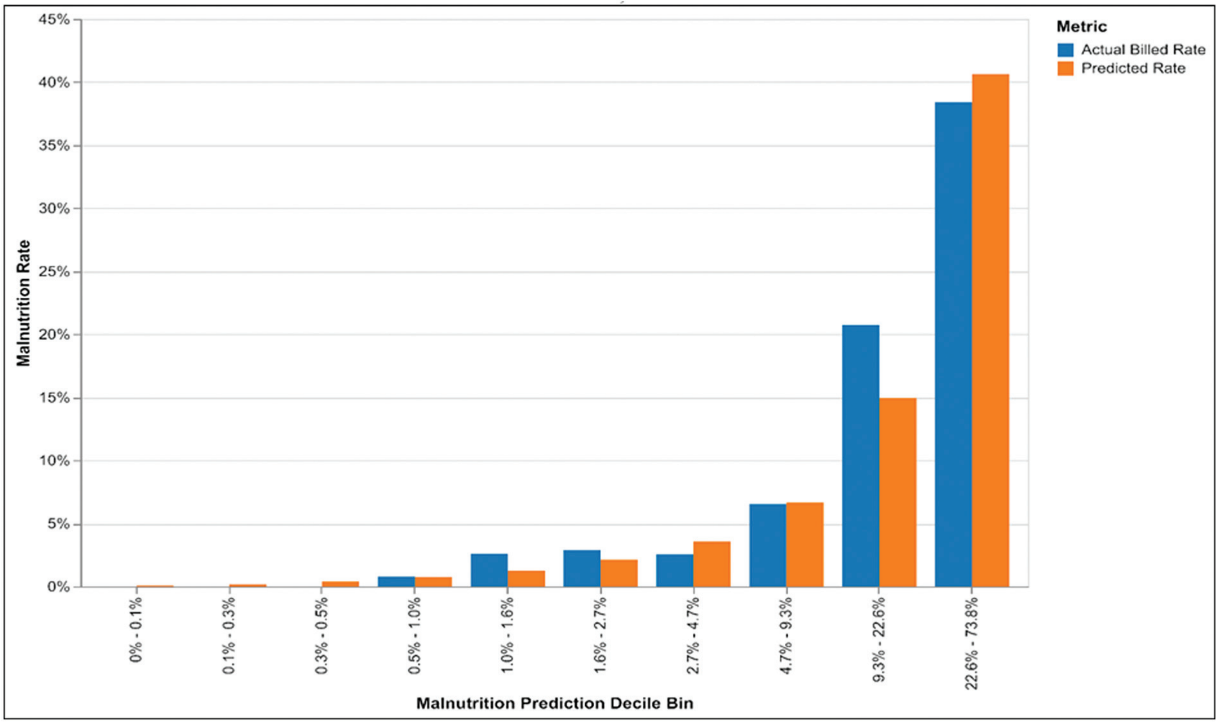


**Supplementary Fig. S10** Actual versus predicted rate of malnutrition patients who reported race as Hispanic. Test set with 2,418 patients who reported race as Hispanic; Actual billed rate: rate of coded malnutrition within each decile; Predicted rate: mean model risk score within each decile.

 © 2025. The Author(s).

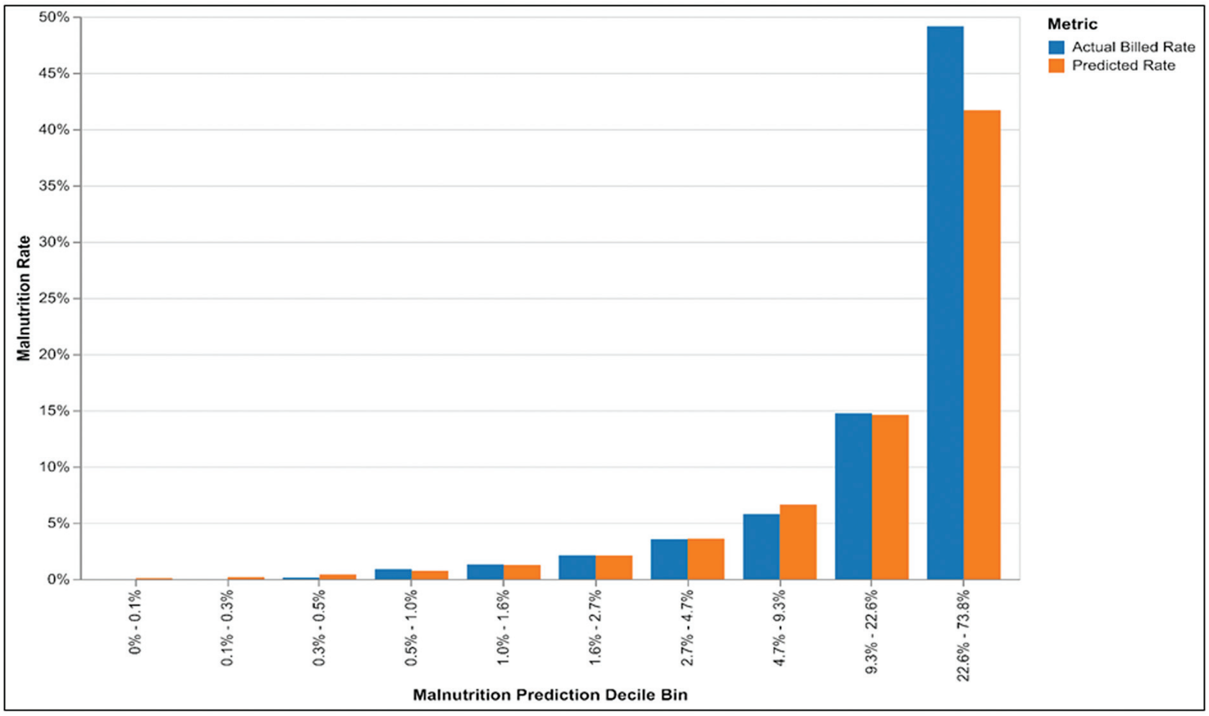


**Supplementary Fig. S11** Actual versus predicted rate of malnutrition patients who reported race as White. Test set with 7,568 patients who reported race as white; actual billed rate, rate of coded malnutrition within each decile; predicted rate, mean model risk score within each decile.

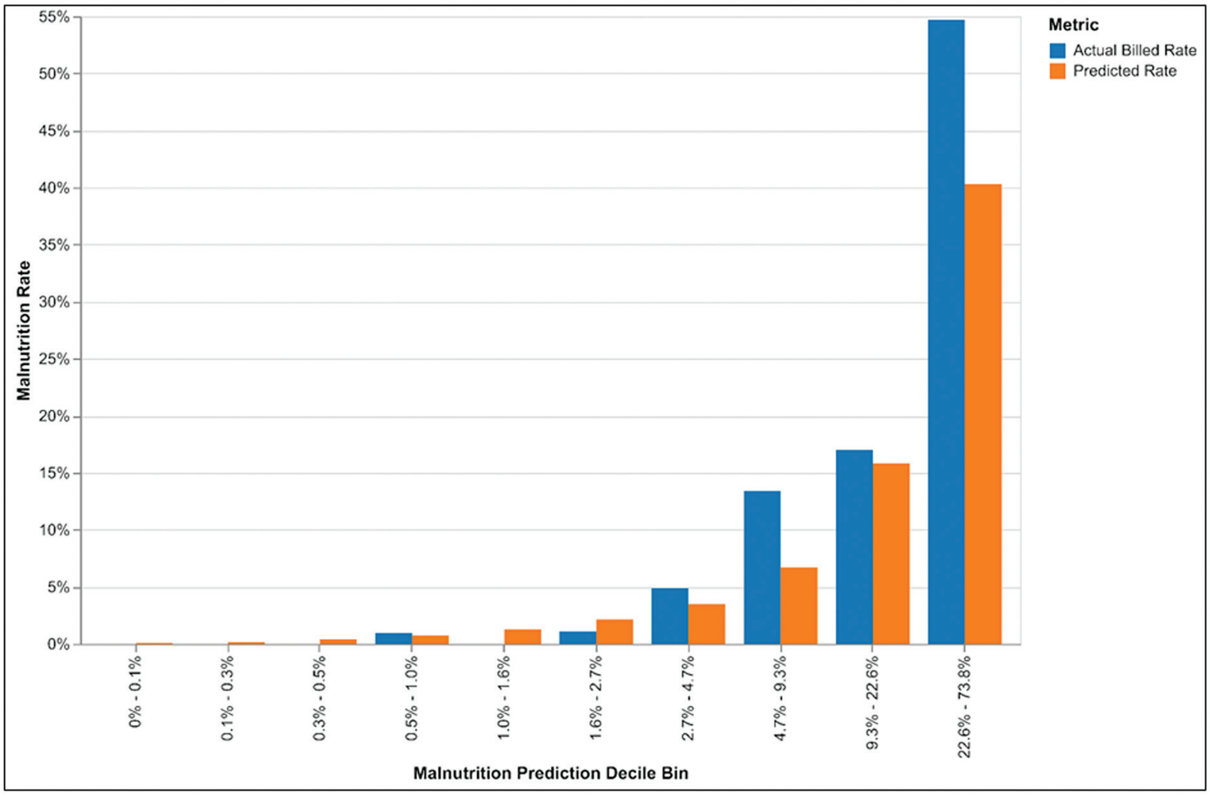


**Supplementary Fig. S12** Actual versus predicted rate of malnutrition patients who reported race as “Other/Unknown/Patient declined.” Test set with 1,030 patients who reported race as Other/Unknown/Patient Declined; actual billed rate, rate of coded malnutrition within each decile; predicted rate, mean model risk score within each decile.

 © 2025. The Author(s).

**Supplementary Table S1** Large language model questions and answers

| Question number | Domain | Question | Answer options |
|---|---|---|---|
| 1 | Anthropometry | Was there any mention of this patient's weight changing over the past month? | Severe loss/moderate loss/mild loss/gain/nothing/unknown |
| 2 | Anthropometry | If the patient has muscle loss, cachexia, or wasting, what is the reported degree? | Mild/moderate/severe/not mentioned/none |
| 3 | Anthropometry | If hand grip strength was measured, what was the patient's result? | Reduced/normal/not mentioned |
| 4 | Anthropometry | If the patient has an amputation, what type do they have? | Below-knee amputation/above-knee amputation/arm/hand/finger/toe/foot/other/multiple/none |
| 5 | Nutrition history | During the past week, what was the adequacy of the patient's nutritional intake? Take into account any notes about nutritional intake at home, PO intake, NPO, enteral feeding, parenteral nutrition, percentage of meals eaten, and whether they were also eating meals or snacks that their family or visitors brought for them. | Poor intake/sufficient intake/unknown/not mentioned |
| 6 | Nutrition history | If the patient has any dietary preferences, intolerances, sensitivities, or allergies, to what extent do these restrict their dietary intake or variety? | Severe/moderate/mild/none |
| 7 | Symptoms | Does the patient have nausea, vomiting, or diarrhea? | Chronic/frequently/currently/previously/none/not mentioned |
| 8 | Symptoms | Does the patient have a poor appetite or anorexia? | Yes/no/not mentioned |
| 9 | Symptoms | Does the patient have physiologic barriers (e.g., nausea, vomiting, odynophagia, or difficulty chewing) to nutrition adequacy? | Severe/moderate/mild/none |
| 10 | Symptoms | What is the level of edema or fluid accumulation? | Mild/moderate/severe/not mentioned/none |
| 11 | Symptoms | Does the patient have frailty or weakness? | No/severe/moderate/mild/not mentioned |
| 12 | Symptoms | What is the patient's physical activity level? | Bedridden/low physical activity/normal physical activity/high physical activity/unknown/not mentioned |
| 13 | Symptoms | Does the patient have signs of inflammation, including fever, leukocytosis, or hypothermia? | Severe/moderate/mild/no/unknown |
| 14 | Medical condition | Does the patient have severe acute inflammation (usually present with critical illness, major infection/sepsis, acute respiratory distress syndrome, severe burns, major abdominal surgery, multitrauma, severe closed head injury, and severe acute pancreatitis)? | Yes/no/unknown |
| 15 | Medical condition | Does the patient have moderate acute inflammation (usually present in chronic diseases complicated by acute moderate exacerbations, or acute new presentations with moderate inflammation associated with Crohn's disease, rheumatologic conditions, chronic obstructive pulmonary disease, pancreatitis, diabetes, infections, and wounds)? | Yes/no/unknown |

(*Continued*)

 © 2025. The Author(s).

**Supplementary Table S1** (*Continued*)

| Question number | Domain | Question | Answer options |
|---|---|---|---|
| 16 | Medical condition | Does the patient have mild to moderate chronic inflammation ("chronic" is when the inflammation lasts for more than 2 weeks)? The inflammation can be confirmed by clinical signs, laboratory markers, or the underlying diagnosis or condition (e.g., congestive heart failure, cystic fibrosis, COPD, Crohn's disease, celiac disease, rheumatoid arthritis, diabetes, abdominal obesity, metabolic syndrome, malignancies, infections like tuberculosis, HIV/AIDS, pressure wounds, periodontal disease, chronic kidney disease, hepatic cirrhosis, mild/moderate pancreatitis, and organ failure or transplant). Inflammation may remit, relapse, or be exacerbated, depending upon the course of disease, treatment modalities or superimposed events or complications. | Yes/no/unknown |
| 17 | Medical condition | Does the patient have a condition that greatly decreases nutritional intake but that has no clear or perceptible inflammation? Examples of such conditions include psychiatric diagnoses like anorexia nervosa and depression; select malabsorptive, obstructive, or dysmotility conditions like esophageal stricture, anatomic short bowel syndrome, and intestinal pseudo-obstruction; and neurological conditions like dysphagia after cerebrovascular accident. | Yes/no/unknown |
| 18 | Psychosocial | Does the patient have a non-disease condition associated with limited resources, or live in an environment that compromises food security, access, or intake (including poverty, famine, and war)? | Yes/no/unknown |
| 19 | Psychosocial | Are there mental illnesses or mental health factors that might affect the patient's nutritional intake? | Yes/no/not documented |
| 20 | Medication | Is the patient taking medications that severely affect appetite, absorption, or metabolism? | Yes/no/unknown/not mentioned |

 © 2025. The Author(s).

**Supplementary Table S2** Accuracy of large language models in benchmarking datasets

| Benchmark | Average | MMLU anatomy | MMLU clinical knowledge | MMLU college biology | MMLU college medicine | MMLU medical genetics | MMLU professional medicine | MedMCQA | MedQA | MedQA-5 options | PubMedQA |
|---|---|---|---|---|---|---|---|---|---|---|---|
| Model | | | | | | | | | | | |
| NousResearch/Meta-Llama-3–8B-Instruct | 67.80% | 70.40% | 72.80% | 74.30% | 63.60% | 77.00% | 72.80% | 55.20% | 59.40% | 55.80% | 76.60% |
| openchat/openchat-3.5–0106 | 63.20% | 63.00% | 71.70% | 76.40% | 66.50% | 68.00% | 71.00% | 50.00% | 53.80% | 46.40% | 65.20% |
| Nexusflow/Starling-LM-7B-β | 63.20% | 63.00% | 69.80% | 75.70% | 64.70% | 69.00% | 72.40% | 50.30% | 53.30% | 46.30% | 67.20% |
| openchat/openchat-3.5–0106-gemma | 62.20% | 59.30% | 68.30% | 71.50% | 64.70% | 69.00% | 66.50% | 49.50% | 53.50% | 46.00% | 73.20% |
| mlabonne/AlphaMonarch-7B | 61.30% | 60.70% | 69.80% | 72.90% | 65.90% | 66.00% | 68.40% | 46.80% | 51.30% | 45.90% | 65.20% |
| NousResearch/Nous-Hermes-2-Mistral-7B-DPO | 61.10% | 51.10% | 68.30% | 72.20% | 64.70% | 68.00% | 66.90% | 49.20% | 52.00% | 45.80% | 72.40% |
| teknium/OpenHermes-2.5-Mistral-7B | 61.10% | 53.30% | 68.70% | 72.20% | 62.40% | 67.00% | 67.60% | 49.60% | 51.20% | 45.10% | 73.40% |
| berkeley-nest/Starling-LM-7B-α | 61.00% | 63.70% | 68.70% | 72.20% | 65.30% | 63.00% | 65.80% | 47.60% | 50.90% | 44.70% | 68.00% |
| NousResearch/Hermes-2-Pro-Mistral-7B | 60.90% | 58.50% | 67.20% | 72.20% | 61.30% | 67.00% | 71.30% | 46.00% | 49.20% | 43.20% | 73.20% |
| yam-peleg/Experiment26–7B | 60.80% | 57.80% | 70.20% | 71.50% | 65.30% | 66.00% | 68.80% | 47.30% | 51.80% | 45.80% | 64.00% |
| lucyknada/microsoft_WizardLM-2–7B | 57.20% | 62.20% | 65.70% | 69.40% | 56.60% | 65.00% | 66.50% | 48.20% | 47.80% | 41.80% | 49.00% |
| mistralai/Mistral-7B-Instruct-v0.2 | 56.80% | 57.80% | 67.90% | 66.00% | 57.20% | 65.00% | 64.70% | 46.20% | 49.00% | 42.90% | 51.20% |
| HuggingFaceH4/zephyr-7b-gemma-v0.1 | 56.00% | 48.10% | 63.80% | 68.10% | 59.00% | 60.00% | 61.80% | 43.00% | 46.70% | 38.50% | 71.20% |
| amazon/MistralLite | 54.30% | 53.30% | 57.70% | 61.80% | 54.30% | 65.00% | 61.00% | 45.90% | 49.80% | 41.40% | 52.40% |
| BioMistral/BioMistral-7B | 51.70% | 48.10% | 60.40% | 56.90% | 56.10% | 60.00% | 57.00% | 43.30% | 44.80% | 37.60% | 52.80% |

Note: Zero-shot performance was employed such that the large language models were not provided with examples, instructional prompts, or feedback on how to answer. Answers that were generated were based on the model's a priori knowledge and reasoning ability.

 © 2025. The Author(s).

**Supplementary Table S3** Accuracy of large language models in QuALITY dataset

| Document length (tokens) | 0–1,000 | 1,000–2,000 | 2,000–3,000 | 3,000–4,000 | 4,000–5,000 | 5,000–6,000 | 6,000–7,000 | 7,000–8,000 | Average |
|---|---|---|---|---|---|---|---|---|---|
| Model | | | | | | | | | |
| openchat/openchat-3.5–0106 | NaN | NaN | 78.60% | 80.10% | 75.40% | 71.40% | 72.60% | 63.80% | 73.70% |
| amazon/MistralLite | NaN | NaN | 79.80% | 78.10% | 73.70% | 69.40% | 71.30% | 66.00% | 73.00% |
| Nexusflow/Starling-LM-7B-β | NaN | NaN | 77.00% | 79.60% | 74.00% | 70.40% | 74.00% | 63.10% | 73.00% |
| NousResearch/Meta-Llama-3–8B-Instruct | NaN | NaN | 80.00% | 82.80% | 72.70% | 72.40% | 65.70% | 62.60% | 72.70% |
| berkeley-nest/Starling-LM-7B-α | NaN | NaN | 77.40% | 78.50% | 74.70% | 69.70% | 66.20% | 62.00% | 71.40% |
| yam-peleg/Experiment26–7B | NaN | NaN | 76.70% | 79.50% | 70.50% | 71.10% | 67.60% | 62.90% | 71.30% |
| NousResearch/Nous-Hermes-2-Mistral-7B-DPO | NaN | NaN | 76.70% | 77.60% | 74.00% | 68.40% | 64.50% | 63.00% | 70.70% |
| mlabonne/AlphaMonarch-7B | NaN | NaN | 75.90% | 79.00% | 70.10% | 69.70% | 65.20% | 62.00% | 70.30% |
| teknium/OpenHermes-2.5-Mistral-7B | NaN | NaN | 75.90% | 77.80% | 72.20% | 68.10% | 64.50% | 62.20% | 70.10% |
| mistralai/Mistral-7B-Instruct-v0.2 | NaN | NaN | 74.30% | 72.50% | 69.00% | 71.10% | 68.60% | 63.50% | 69.80% |
| NousResearch/Hermes-2-Pro-Mistral-7B | NaN | NaN | 76.70% | 76.20% | 69.80% | 68.10% | 64.50% | 60.50% | 69.30% |
| lucyknada/microsoft_WizardLM-2–7B | NaN | NaN | 68.10% | 71.20% | 68.00% | 63.50% | 60.80% | 54.80% | 64.40% |
| BioMistral/BioMistral-7B | NaN | NaN | 61.10% | 56.80% | 51.20% | 51.60% | 48.00% | 42.50% | 51.90% |
| Average | | | 73.93% | 71.54% | 68.04% | 64.80% | 65.23% | 65.02% | |

Zero-shot performance was employed such that the large language models were not provided with examples, instructional prompts, or feedback on how to answer. Answers that were generated were based on the model's a priori knowledge and reasoning ability.

 © 2025. The Author(s).

**Supplementary Table S4** Comparison of machine learning models with and without large language model features included

| Risk of malnutrition on the first day of hospitalization | | | | | | |
|---|---|---|---|---|---|---|
| Model combination | Sensitivity | Specificity | PPV | NPV | AUROC | AUPRC |
| LLM and other features | 0.4893 | 0.9673 | 0.5516 | 0.9584 | 0.9152 | 0.5540 |
| LLM only | 0.2988 | 0.9770 | 0.5170 | 0.9443 | 0.8249 | 0.3751 |
| Other features only | 0.4482 | 0.9657 | 0.5178 | 0.9551 | 0.9054 | 0.5020 |
| **Maximum risk of malnutrition during hospitalization** | | | | | | |
| Model combination | Sensitivity | Specificity | PPV | NPV | AUROC | AUPRC |
| LLM and other features | 0.5845 | 0.9749 | 0.6569 | 0.9661 | 0.9529 | 0.6700 |
| LLM only | 0.1410 | 0.9939 | 0.6537 | 0.9336 | 0.8249 | 0.3751 |
| Other features only | 0.5668 | 0.9727 | 0.6303 | 0.9647 | 0.9486 | 0.6411 |

Abbreviations: AUPRC, area under the precision–recall curve; AUROC, area under the receiver-operating characteristic curve; LLM, large language model; NPV, negative predictive value; PPV, positive predictive value.
Risk threshold on first day of hospitalization: 41.50%. Maximum risk threshold throughout hospitalization: 67.50%.

**Supplementary Table S5** Ethnic group and race of hospital admissions

| Ethnic group and race (*n*, %) | Total | Malnourished | Not malnourished | *p*-Value for difference |
|---|---|---|---|---|
| Ethnic Group: Hispanic; Race: Black or African American | 207 (0.2%) | 0 (0.0%) | 196 (0.2%) | $p < 0.0001$ |
| Ethnic Group: Hispanic; Race: Other | 4,234 (4.0%) | 232 (3.2%) | 4,002 (4.0%) | $p = 0.0003$ |
| Ethnic Group: Hispanic; Race: White | 12,416 (11.7%) | 846 (11.6%) | 11,570 (11.7%) | $p = 0.9547$ |
| Ethnic Group: Non-Hispanic; Race: American Indian or Alaska Native | 209 (0.2%) | 0 (0.0%) | 197 (0.2%) | $p < 0.0001$ |
| Ethnic Group: Non-Hispanic; Race: Asian | 8,249 (7.7%) | 626 (8.6%) | 7,623 (7.7%) | $p = 0.0052$ |
| Ethnic Group: Non-Hispanic; Race: Black or African American | 14,652 (13.8%) | 1,167 (16.0%) | 13,485 (13.6%) | $p < 0.0001$ |
| Ethnic Group: Non-Hispanic; Race: Native Hawaiian or Other Pacific Islander | 325 (0.3%) | 0 (0.0%) | 300 (0.3%) | $p < 0.0001$ |
| Ethnic Group: Non-Hispanic; Race: Other | 4,732 (4.4%) | 322 (4.4%) | 4,410 (4.4%) | $p = 0.9765$ |
| Ethnic Group: Non-Hispanic; Race: Patient declined | 114 (0.1%) | 0 (0.0%) | 106 (0.1%) | $p = 0.0014$ |
| Ethnic Group: Non-Hispanic; Race: Unknown | 151 (0.1%) | 0 (0.0%) | 139 (0.1%) | $p = 0.0001$ |
| Ethnic Group: Non-Hispanic; Race: White | 58,849 (55.3%) | 3,758 (51.7%) | 55,091 (55.5%) | $p < 0.0001$ |
| Ethnic Group: Patient declined; Race: Patient declined | 145 (0.1%) | 0 (0.0%) | 140 (0.1%) | $p < 0.0001$ |
| Ethnic Group: Patient declined; Race: White | 159 (0.1%) | 0 (0.0%) | 150 (0.2%) | $p < 0.0001$ |
| Ethnic Group: Unknown; Race: Unknown | 1,059 (1.0%) | 173 (2.4%) | 886 (0.9%) | $p < 0.0001$ |
| Ethnic Group: Unknown; Race: White | 298 (0.3%) | 0 (0.0%) | 271 (0.3%) | $p < 0.0001$ |

 © 2025. The Author(s).

**Supplementary Table S6** Summary of model variations, configurations, and key differences in predictive performance

| Model variations | Risk threshold configurations | Overall key differences in comparison to M-MST |
|---|---|---|
| Malnutrition risk on the first day of admission, indexed against hospital-coded malnutrition (►Table 2) | Intensive care unit: 65.1%; Non-intensive care unit: 39.7%; Overall: 42.2% | Improved sensitivity, specificity, PPV, and NPV, with the greatest improvements in sensitivity and PPV |
| Maximum malnutrition risk throughout hospitalization-indexed against coded malnutrition (►Table 2) | Intensive care unit: 85.5%; Non-intensive care unit: 65.4%; Overall: 68.1% | Improved sensitivity, specificity, PPV, and NPV, with the greatest improvements in sensitivity and PPV |
| Malnutrition risk on the first day of admission, indexed against dietitian-recorded malnutrition (►Table 2) | Intensive care unit: 65.1%; Non-intensive care unit: 39.7%; Overall: 42.2% | Improved sensitivity and PPV |
| Maximum malnutrition risk throughout hospitalization indexed against dietitian-recorded malnutrition (►Table 2) | Intensive care unit: 85.5%; Non-intensive care unit: 65.4%; Overall: 68.1% | Improved sensitivity and PPV |
| 30-, 60-, 90-day hospital mortality and readmission rates (►Table 3) | Risk threshold 42.2% | Improved ability to identify patients at risk for 30-, 60-, and 90-day hospital mortality and readmission, with the greatest difference seen with mortality |

Abbreviations: M-MST, nurse-administered, modified version of the Malnutrition Screening Tool; NPV, negative predictive value; PPV, positive predictive value.
For comparisons between the model and the M-MST, the model's risk threshold is adjusted such that it identifies the same number of patients at risk of malnutrition as the M-MST. For mortality and readmission, statistical significance tests were performed on only the non-overlapping observations (those flagged by both model and M-MST were excluded).

**Supplementary Table S7** Agreement between discharge-coded malnutrition and dietitian-reported malnutrition

| | | Discharge-coded malnutrition | | |
|---|---|---|---|---|
| | | **No** | **Yes** | **Total** |
| Dietitian-recorded malnutrition | No | 97,228 (91.3%) | 4,255 (4.0%) | 101,483 (95.3%) |
| | Yes | 1,948 (1.8%) | 3,018 (2.8%) | 4,966 (4.7%) |
| | Total | 99,176 (93.2%) | 7,273 (6.8%) | 106,449 |

 © 2025. The Author(s).
